# Disinfection of sink drains to reduce a source of three opportunistic pathogens, during *Serratia marcescens* clusters in a neonatal intensive care unit

**DOI:** 10.1371/journal.pone.0304378

**Published:** 2024-06-12

**Authors:** Thibault Bourdin, Marie-Ève Benoit, Michèle Prévost, Dominique Charron, Caroline Quach, Eric Déziel, Philippe Constant, Emilie Bédard

**Affiliations:** 1 INRS-Armand-Frappier Santé Biotechnologie, Laval, QC, Canada; 2 CHU Sainte-Justine Research Center, Montréal, QC, Canada; 3 Polytechnique Montréal, Montréal, QC, Canada; 4 CHU Sainte-Justine, Université de Montréal, Montréal, QC, Canada; VIT University, INDIA

## Abstract

**Objective:**

Evaluate the effects of five disinfection methods on bacterial concentrations in hospital sink drains, focusing on three opportunistic pathogens (OPs): *Serratia marcescens*, *Pseudomonas aeruginosa* and *Stenotrophomonas maltophilia*.

**Design:**

Over two years, three sampling campaigns were conducted in a neonatal intensive care unit (NICU). Samples from 19 sink drains were taken at three time points: before, during, and after disinfection. Bacterial concentration was measured using culture-based and flow cytometry methods. High-throughput short sequence typing was performed to identify the three OPs and assess *S*. *marcescens* persistence after disinfection at the genotypic level.

**Setting:**

This study was conducted in a pediatric hospitals NICU in Montréal, Canada, which is divided in an intensive and intermediate care side, with individual rooms equipped with a sink.

**Interventions:**

Five treatments were compared: self-disinfecting drains, chlorine disinfection, boiling water disinfection, hot tap water flushing, and steam disinfection.

**Results:**

This study highlights significant differences in the effectiveness of disinfection methods. Chlorine treatment proved ineffective in reducing bacterial concentration, including the three OPs. In contrast, all other drain interventions resulted in an immediate reduction in culturable bacteria (4–8 log) and intact cells (2–3 log). Thermal methods, particularly boiling water and steam treatments, exhibited superior effectiveness in reducing bacterial loads, including OPs. However, in drains with well-established bacterial biofilms, clonal strains of *S*. *marcescens* recolonized the drains after heat treatments.

**Conclusions:**

Our study supports thermal disinfection (>80°C) for pathogen reduction in drains but highlights the need for additional trials and the implementation of specific measures to limit biofilm formation.

## 1. Introduction

Each year, millions of hospitalizations are complicated by healthcare-associated infections (HAIs) [1], which increase morbidity and mortality rates, prolong hospital stays, and substantially inflate the cost of medical care [2–5]. Newborns in neonatal intensive care units (NICUs) are a vulnerable population at increased risk due to their low birth weight, prematurity, and exposure to numerous invasive procedures [6–8]. Over the last few decades, HAIs have emerged as a significant global burden, exacerbated by the alarming increase in multidrug-resistant pathogens. In response, implementing robust infection prevention and control measures within hospital settings has become imperative. One prominent source of microorganisms in healthcare facilities is the sink drain, which may act as a reservoir due to the presence of biofilm harbouring waterborne opportunistic pathogens (OPs) [9–14]. Numerous nosocomial outbreaks of bacterial pathogens have been linked to sink drains located in patient rooms [3, 6, 12, 15–21]. When individuals wash their hands or pour liquids into the sink, splashing is a common occurrence, especially near the drain [3, 22–25]. This event leads to potential contamination of material and surfaces in close proximity, as well as skin or clothes of nearby patients and healthcare personnel. Moreover, these splashes can generate aerosols containing potentially harmful contaminants in the surrounding air [12, 26, 27], posing a risk of inhalation by patients.

Cleaning and disinfection are essential strategies to reduce bacterial load in drains and to remove OPs involved in outbreaks. The effectiveness of disinfection relies on several factors, including the type of disinfectant, its concentration, the exposure time, the frequency of application, and the tolerance of biofilm-associated bacteria to the disinfectant. Biofilms provide a protective environment for bacteria [28, 29], making the exposure time and the action mode crucial for ensuring effective penetration of the disinfectant. Employing foam instead of liquid products or using specialized devices that retain disinfectants in the P-Trap can lead to longer exposure times, resulting in reduced bacterial load in drains [30–34].

Various disinfectants have been tested with limited success in decreasing the bacterial load in drains, such as chlorine [35], steam [16], acetic acid [36, 37], ozonated water [34] and hydrogen peroxide [38–40]. However, if a single treatment is carried out, OPs typically reappear in the drain a few days later [16, 33, 38, 41]. Therefore, establishing a recurrent cleaning and disinfection routine is essential to prevent the resurgence of OPs in sink drains after an outbreak. A more expensive, but apparently more effective alternative is to install self-disinfecting drain devices that can generate high temperatures, vibrations, and/or emit UV rays to prevent biofilm formation [18, 27, 42].

Despite the various studies on waterborne OPs outbreaks, there is currently no consensus regarding the most effective measure to control their presence in real drain biofilms. In many cases, combining different strategies has proven to be more effective, but the impact of a single intervention is often challenging to measure [15, 30, 43–48]. Therefore, more evidence is needed to demonstrate the effectiveness of drain disinfection in limiting OPs transmission in hospitals, and a clear disinfection protocol must be established.

The aim of this study was to assess the efficacy of various treatments in reducing bacterial loads in hospital sink drains, and eliminating waterborne OPs: *S*. *marcescens*, *Stenotrophomonas maltophilia* and *Pseudomonas aeruginosa*. These findings will help to prevent pathogen transmission from sinks to patients. The study was conducted in a NICU where multiple *Serratia marcescens* outbreaks occurred between 2019 and 2022.

## 2. Materials and methods

This study was conducted in the NICU of a 417 beds pediatric hospital in Montréal, Canada. The unit is divided in two sections: the intensive care with 35 beds and the intermediate care with 45 beds. Patients are in individual rooms, each room having its own sink. The stainless-steel sink is connected to a chrome-plated brass drain and has dual foot pedals for the activation of hot and cold water at the tap. The water jet and drain are not aligned.

### 2.1 Sink drains interventions

Five drain disinfection treatments were evaluated, including self-disinfecting drains, chlorine disinfection, boiling water disinfection, hot tap water flushing, and steam disinfection. The interventions were carried out according to the timeline presented in section 2.2.

#### Self-disinfecting drain

KLEANIK™ Sink Disinfection System (Surgmed Group, Montreal, QC, Canada) was used (S1 Fig). This device helps to prevent biofilm formation in sink drain, using multiple vibration cycles (≥ 50 Hz) and high temperature disinfection by heating drain water to at least 80°C.

#### Chlorine and boiling water disinfection

To extend the exposure time of the drain’s biofilm to the disinfectant, a stop valve was installed between the P-Trap outlet and the wall (S1 Fig). The cost of installing valve is approximately 400 to 500$CAD, which includes the cost of the valve (100$CAD), the cost of accessories needed to adapt the valve to the drain and the cost of labor. When the valve was closed, the disinfectant was retained in the drain, allowing for an extended and controlled exposure time. With the use of a stop valve, higher contact times can be provided thus allowing the use of lower concentrations of chlorine, limiting degassing and material corrosion issues. During the disinfection process, 500 mL of a chlorine solution (20 ppm) was initially poured down the drain to displace the volume of water always present in the bottom of the P-Trap between uses to avoid odor problems. Subsequently, the drain valve was closed, and the chlorine solution was poured into the drain until it reached the strainer (approximately 1500 mL). A flat rubber plug was then placed over the strainer during the disinfection period to avoid exposure with the ambient air.

The same approach was employed with boiling water (≥ 90°C) disinfection, except that drains were first warmed up with hot tap water for five minutes. During disinfection, the drain was wrapped with an insulating material to prevent temperature loss due to the drain wall exposure to ambient temperature.

An exposure time of 30 minutes was selected for chlorine and boiling water, as it was considered to be a reasonable duration for sink maintenance. To better understand the factors affecting the effectiveness of the disinfection, boiling water initial and final water temperatures in drains were measured using a thermometer (Omega Engineering, Norwalk, CT, USA), while initial and final residual chlorine concentrations were measured using a pocket colorimeter (HACH, Loveland, CO, USA).

#### Hot tap water flushing

Hot tap water was flushed down the drain for 30 consecutive minutes once a stable temperature was reached. A towel was used to cover the sink during the treatment to prevent the spread of aerosols.

#### Steam disinfection

To initiate the steam disinfection process, the hose of the steam disinfection system was placed inside the drain and secured to the strainer using a rubber plug (S1 Fig). The disinfection procedure was conducted for 3 minutes at 120°C. Subsequently, hot water from the faucet was allowed to flow freely for 1 minute, flushing out any residual biofilm in the drain. To maintain a safe and controlled environment, a towel was placed over the sink during the entire disinfection process to prevent the spread of aerosols.

### 2.2 Sampling campaigns

**Fig 1:**
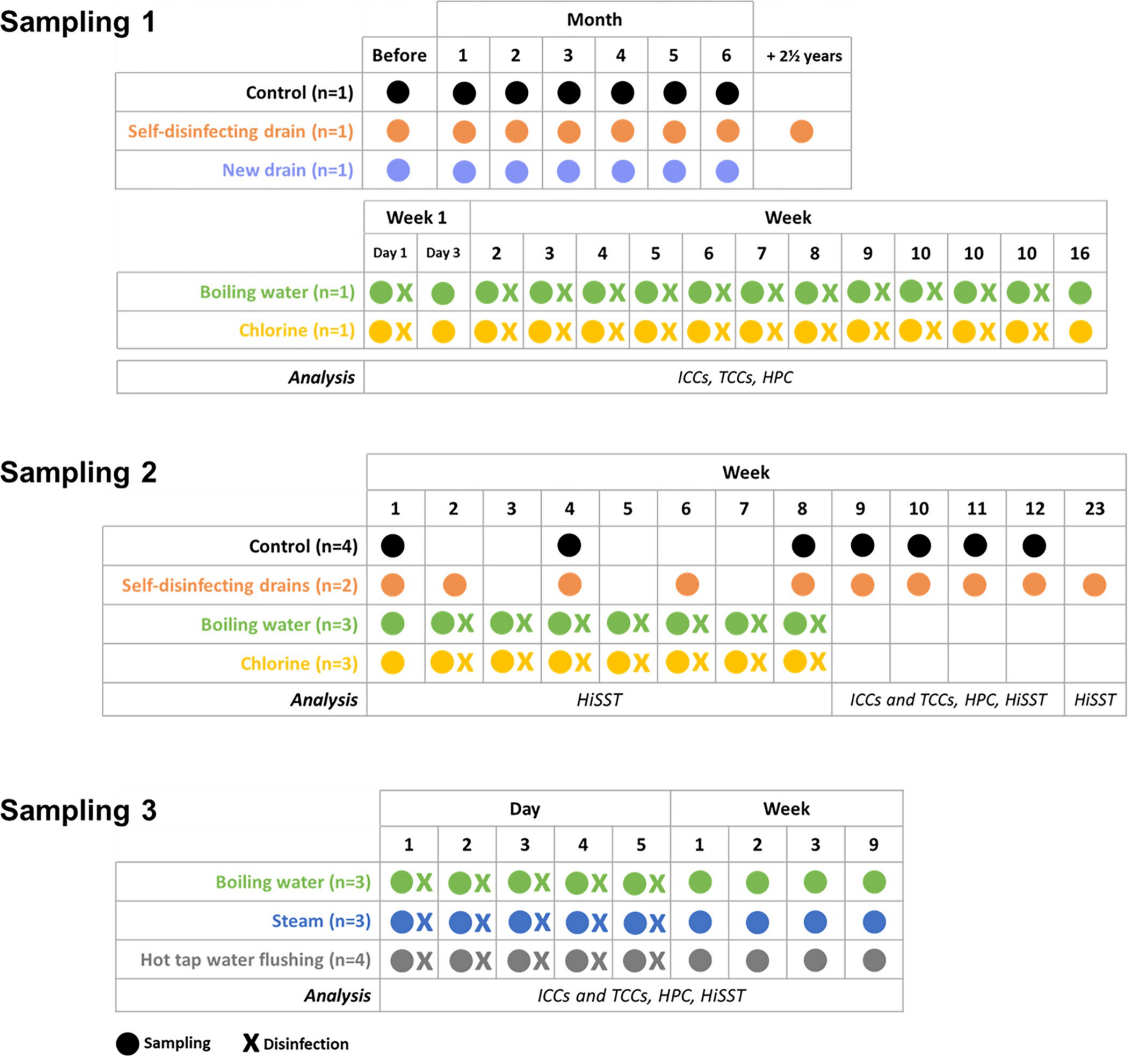
Sampling and disinfection timeline of sampling campaigns. Four interventions were carried out in the first sampling; the comparison between thermal disinfection and other disinfection methods was done during the second sampling; and the third sampling was used to compare the different thermal disinfection methods. Circles represent the sampling dates and “X” symbols represent disinfection dates. Sampling was always conducted prior to disinfection when both are recorded on a given day. Bacterial loads were measured with flow cytometry for intact cell counts (ICCs) and total cell counts (TCCs), and with heterotrophic plate count (HPC). OPs were detected by High-Throughput Short Sequence Typing (HiSST).

During three sampling campaigns between 2019 and 2021 (Fig 1), collected samples were processed for total (TCCs) and intact (ICCs) cell counts, using flow cytometry, heterotrophic plate count (HPC), and PCR analyses, or archived at -80°C. A total of 100 mL drain water was sampled using a syringe connected to a sterile 3.1 mm Neoprene® flexible tube. Biofilm from the inner sides of sink drains was sampled using a nylon-flocked swab (Puritan Medical Products, Guilford, ME, USA) fixed on a sterile wooden extension, swabbing the drain from the top of the strainer to the bottom of the P-Trap. Swabs were then placed in 15 mL sterile tubes with 2 mL of sterile saline water. Samples were collected on the treatment day, just before performing the treatment.

#### Testing of four drain interventions (sampling campaign 1)

The first step was to conduct four distinct drain interventions. The effects of installing a self-disinfecting drain and a new P-Trap on bacterial concentrations in the drain were evaluated on two different patient room sinks, and then compared to a control drain. The installation of a new P-Trap consisted of replacing all parts of the drain (tailpiece, trap and wall tube) except the strainer, with new ones. Chlorine and boiling water disinfection were also tested on two handwashing stations outside patient rooms. Sink drains were sampled, disinfected, and subjected to analysis according to the sampling and disinfection timeline described in Fig 1.

#### Comparison of self-disinfection, chlorine, and boiling water disinfection methods (sampling campaign 2)

A year and a half after the initial sampling campaign (sampling 1), three interventions were chosen for further examination during the second sampling campaign: self-disinfecting drains (n = 2), chlorine disinfection (n = 3), and boiling water disinfection (n = 3). Additionally, control drains (n = 4) without intervention were sampled. Two new self-disinfecting drains, different from the one used during the first sampling campaign, had been installed in patient rooms a year and a half earlier. The impact of device shut down was studied by disconnecting the self-disinfecting drains for 3 weeks (week 5 to 7) and measuring the three OPs present in drains. Both chlorine and boiling water disinfection methods were applied to two patient sinks, and the same handwashing sinks that had been previously disinfected during the first sampling campaign, using the same treatment methods. Sink drains were sampled, disinfected, and subjected to analysis according to the sampling and disinfection timeline described in Fig 1.

#### Comparison of thermal disinfection methods (sampling campaign 3)

A third disinfection campaign compared three different methods of thermal disinfection of patient room sink drains, including boiling water (n = 3), steam (n = 3), and hot tap water flushing (n = 4). Two of the three sink drains used for boiling water disinfection were from patient room sinks that had previously undergone disinfection with boiling water during the second sampling campaign. The third drain belonged to the hand-washing station and had been subjected to chlorine disinfection during both the first and second sampling campaigns. A seven-week interval elapsed between the second and third sampling campaigns. Drains were sampled, disinfected, and analyzed according to the sampling and disinfection timeline described in Fig 1.

### 2.3 Sample analysis

Drain biofilm samples from swabs were thoroughly mixed (vortex) during 30 seconds at maximum speed after the addition of 4–5 sterile glass beads to the sample. Biofilm suspensions (1–2 mL) were then added to the corresponding drain water sample (100 mL). Nearly half of the mixture (50 mL) was used for bacterial count analysis and the other half for OPs detection. To perform bacterial counts, samples were diluted with saline water to the appropriate dilutions. Results are reported per mL of drain sample.

#### Heterotrophic plate counts

For each sample, 1 mL of sample or its dilution was filtered on 0.45 μm pore size mixed cellulose ester (MCE) sterile membranes (Millipore Sigma-Aldrich, Oakville, ON, Canada), which were placed on R2A agar plates (Becton Dickinson, Franklin Lakes, NJ, USA). Duplicate plates were incubated at room temperature for 7 days [49], and the resulting colony-forming units (CFU) were counted to quantify HPC.

#### Total and intact cell counts

Flow cytometry was used to quantify total cell counts (TCCs) and intact cell counts (ICCs). In a 96-well plate, 300 μL of the samples were added and incubated at 37°C for 3 minutes. For each sample dual staining methods was performed in duplicates. For the first staining method, 3 μL of SYBRGreen1 (MilliporeSigma Canada Ltd, Oakville, ON, CA) was used for TCCs. For the second staining method, 3 μL of a mixture of SYBRGreen and propidium iodide (Molecular Probes, Eugene, OR, USA) was used for ICCs. The plate was then incubated at 37°C for 10 minutes. A BD Acuri-C6 flow cytometer (Becton Dickinson, Franklin Lakes, NJ, USA) was used with a 488 nm argon laser. FL1 and FL3 filters were selected with a threshold of 800 in FL1 and a fast flow (66 μL/min). Data was analysed with the BD CSampler software [50].

#### Opportunistic pathogen detection

A detailed description of subsequent sample processing, quality control of the filtrations, and DNA extraction procedures is provided in our previous study [51]. Briefly, drain water samples (50 mL) dedicated to HiSST genotyping were filtered on 0.22 μm MCE sterile membranes. The DNA from genomic environmental samples was extracted prior to PCR amplicon sequencing. DNA extraction procedure combined mechanical and chemical lysis, using a bead beater and ammonium acetate treatment [51]. The DNA extract was quantified (NanoDrop™ 2000c, Thermo Fisher Scientific), diluted to 25 ng/μL and stored at -80°C. *S*. *marcescens* was detected by PCR targeting the locus *bssA* comprised in the HiSST-scheme developed for *S*. *marcescens* genotyping [52]. *P*. *aeruginosa* and *S*. *maltophilia* detection were performed by PCR targeting loci *pheT* and *glnG*, respectively, using primers described previously [53].

The typing of *S*. *marcescens* species was studied specifically due to multiple colonization events that occurred during five clusters reported in this NICU [51]. Additional analyses were performed to verify the distribution of *S*. *marcescens* genotypic profiles in sink drain samples, following the HiSST procedure previously described [51]. HiSST analysis allowed to verify the presence of the same *S*. *marcescens* genotypes at different sampling dates. This provided insight on the nature of the recolonization, whether regrowth of previously present genotypes (endogenous recolonization) or acquisition of new strains (exogenous source). When a genotype remained in the sink drain after the intervention, we considered this as a suggestion of some inefficiency of the methods; else, a one-time presence of a genotype after drain disinfection was considered as the difficulty of the strain to maintain itself in the drain. Briefly, the HiSST method consists of amplifying specifically three high discriminatory loci (242–318 bp), using primers developed for the *S*. *marcescens* HiSST scheme which include Illumina linker sequences [52]. PCR products were subjected to library preparation that allows the combination of several samples at equimolar concentration, and by adding barcoded primers (S1 Table) supplied by Integrated DNA Technologies Inc. (Mississauga, ON, Canada). Libraries were sequenced using the Illumina MiSeq PE-250 platform at the Centre d’Expertise et de Services Génome Québec (Montréal, QC, Canada). All steps of raw sequencing read processing were performed using the HiSST-dada2 pipeline, on R environment [54, 55], available on Github at https://github.com/LaboPC/HiSST-schemes_TB. The proportion of reads remaining after each step of the bioinformatics pipeline is provided in S1 Table.

### 2.4 Statistical analysis

The mean of biological replicates, which were samples collected from multiple sink drains subjected to the same treatment, was employed for each treatment date during the assessment of treatment effects on HPCs and ICCs. The non-parametric Mann-Whitney U test and Wilcoxon signed-rank test were used to compare differences in means. Statistically significant differences were identified if the p-value was less than 0.05. Analysis were performed through R environment [55].

### 2.5 Accession number

Raw sequencing reads have been deposited in the Sequence Read Archive of the NCBI in the BioProject PRJNA1030081.

## 3. Results

### 3.1 Effect of treatments on HPCs and ICCs

#### Impact of P-Trap replacement and of self-disinfecting drains (sampling 1 and 2)

**Fig 2:**
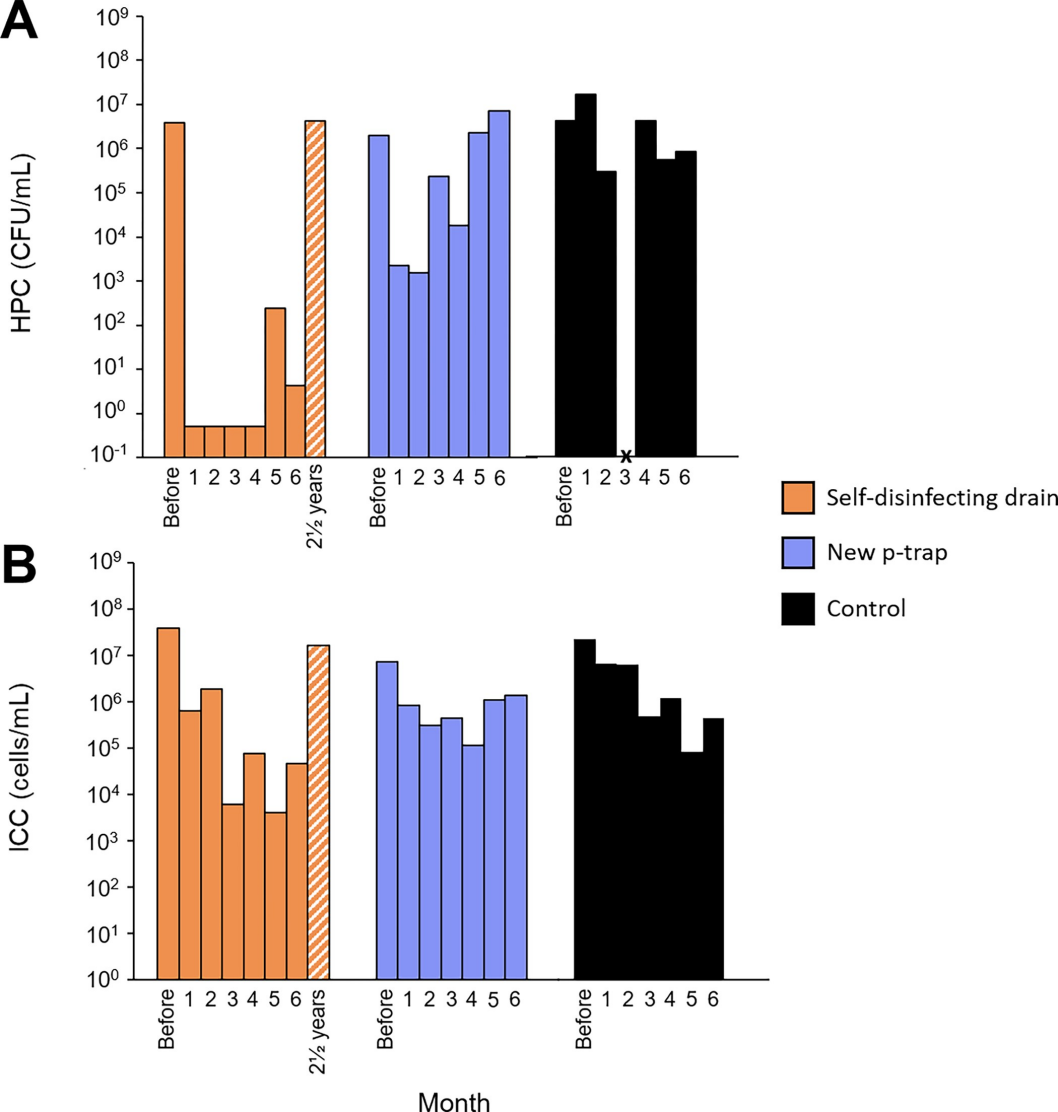
Concentrations of (A) heterotrophic plate count (HPC) and (B) flow cytometry intact cell count (ICC) in drains before and after the installation of a self-disinfecting drain and a new P-Trap. Drains were sampled once a month for 6 months. Bars represent the mean of replicates for each sample. CFU = Colony-forming units. Missing data is represented by “X”.

During the first month following the replacement of the original sink drain with a self-disinfecting drain, HPCs remained 7-log below the initial pre-change level (3.85 x 10^6^ CFU/mL, Fig 2). Over the following four months, no culturable bacteria were detected. By month six, HPCs increased to 4.4 CFU/mL. ICCs decreased more progressively over the months, reaching 4.50 x 10^4^ cells/mL at month six, equivalent to a reduction of almost 3-log from initial value. Two and a half years after its installation and continuous operation, HPCs and ICCs measured in the self-disinfecting drain were back to their initial values (Fig 2). Replacement of the P-trap resulted in a 3-log decrease in HPC compared with pre-change levels. A rapid recolonization of the drain occurred, reaching the concentration detected prior to the P-Trap replacement, within 5 months. The replacement of the P-Trap decreased the ICCs of 1-log, which remained stable throughout the six-month period. TCCs followed the same trends observed for ICCs (S2 Fig). Throughout the same sampling period, the control drain had varying HPC, ICC and TCC concentrations (mean ± SD, 4.44 x 10^6^ ± 6.18 x 10^6^ CFU/mL, 5.24 x 10^6^ ± 7.83 x 10^6^ cells/mL and 7.74 x 10^7^ ± 1.28 x 10^8^ cells/mL, respectively).

During the second sampling campaign, two self-disinfecting drains were sampled in parallel with control drains once a week for five weeks. During those weeks, HPC and ICC concentrations in the self-disinfecting drains remained lower than that observed in the control drains, even one and a half year after their installation (S3 Fig). Concentrations did not reach the values observed in the self-disinfecting drain of the first sampling campaign when it was sampled two and a half year after its installation. HPCs, ICCs and TCCs in these control drains (mean ± SD, 2.56 x 10^6^ ± 1.44 x 10^6^ CFU/mL, 6.47 x 10^6^ ± 4.51 x 10^6^ cells/mL and 6.02 x 10^7^ ± 3.93 x 10^7^ cells/mL, respectively) are comparable to the concentration in the control drain sampled during the first sampling campaign.

#### Impact of boiling water and chlorine disinfection (sampling campaign 1)

**Fig 3:**
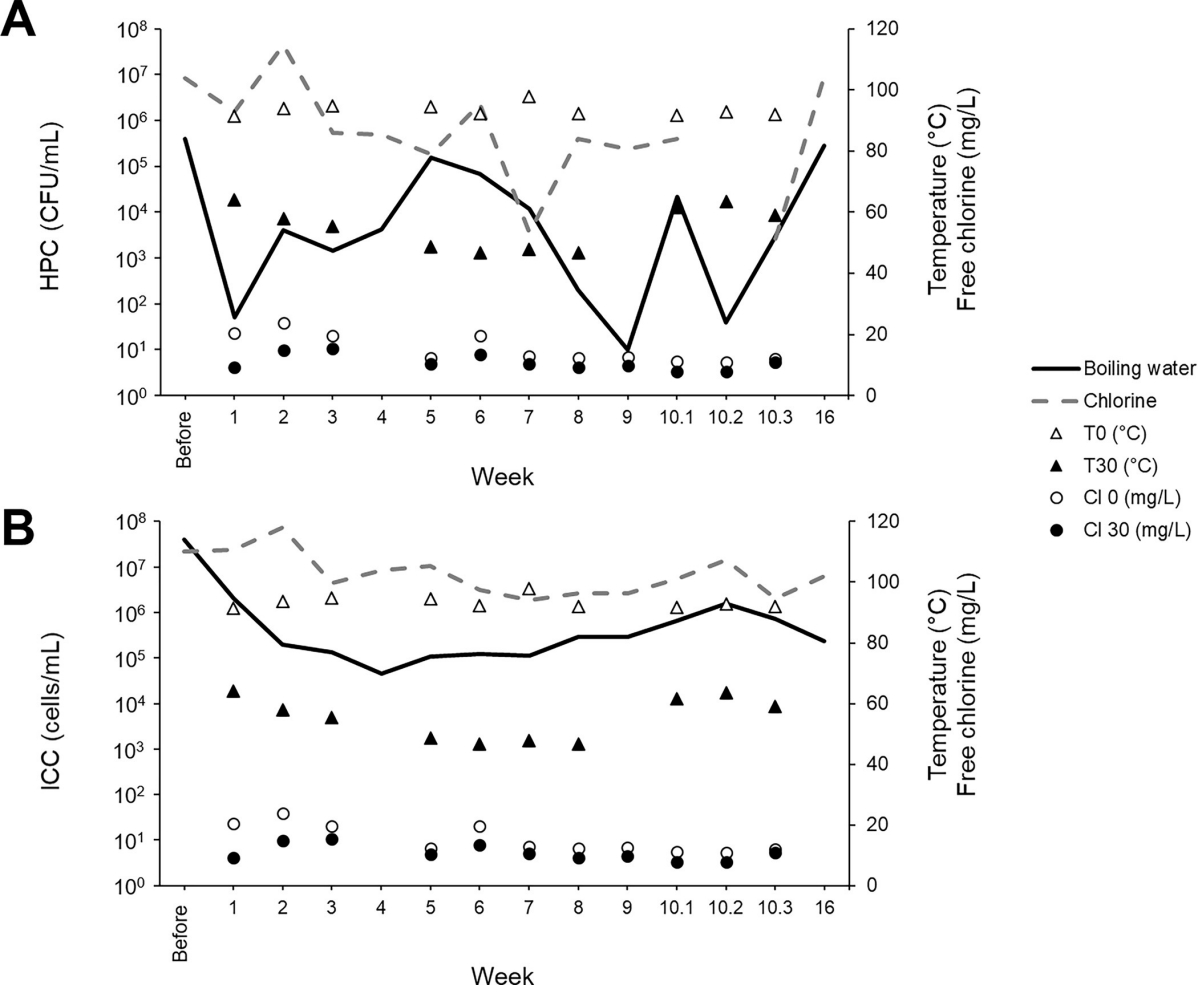
Concentrations of (A) heterotrophic plate counts (HPCs) and (B) intact cell counts (ICCs) in drains before, during and after chlorine and boiling water disinfection. Boiling water results are represented by the full line, and chlorine results by the dotted line. Initial (T0, unfilled triangles) and final (T30, filled triangles) temperatures were measured for the boiling water disinfection as well as the initial (Cl 0, unfilled circles) and final (Cl 30, filled circles) free chlorine for the chlorine disinfection. Colony-forming units = CFU.

Fig 3 shows the impact of weekly disinfection with boiling water and chlorine at two handwashing stations for 10 weeks. For the boiling water disinfection, a reduction of HPCs (4-log) was observed in the 48 hours after disinfection. A notable reduction by 3-log was measured for ICCs after 4 weeks of boiling water disinfection, followed by a slight increase ranging from 1.09 x 10^5^ and 1.60 x 10^6^ cells/mL. This increase follows an upward trajectory, potentially indicating a gradual adaptation of the population beginning at week 4. Chlorine disinfection was somewhat effective for HPCs (1-log) and less effective for ICCs, with concentrations stabilizing between 1.91 x 10^6^ and 7.22 x 10^7^ cells/mL. On week 10, drains were sampled and disinfected on 3 different days (10.1, 10.2 and 10.3 on Fig 3). Finally, drains were also sampled six weeks after the end of the disinfection (week 16). Interestingly, HPC concentrations returned to their initial value for both disinfection methods. However, ICCs remained 2-log below the initial value after boiling water disinfection, while TCCs followed the same trend observed for ICCs (S4 Fig).

#### Impact of hot tap water flushing, boiling water disinfection and steam injection (sampling campaign 3)

**Fig 4:**
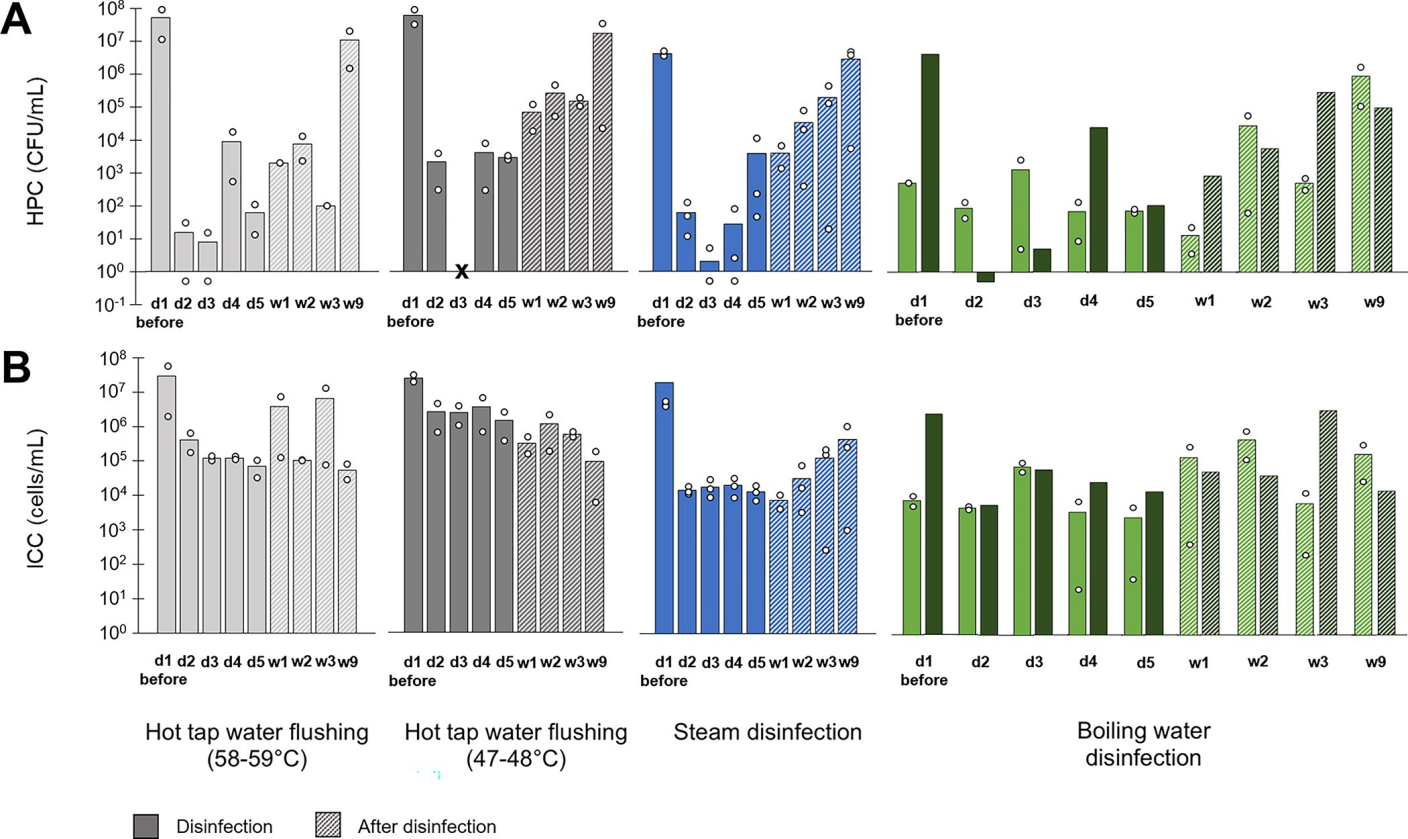
Concentrations of (A) heterotrophic plate count (HPC) and (B) flow cytometry intact cell count (ICC) in drains during and after thermal disinfection. Bars represent the mean of replicates for each sample (symbolized by empty circle) from sinks with hot tap water flushing (n = 2 at 58–59°C; and n = 2 at 47–48°C), steam disinfection (n = 3) and boiling water disinfection (n = 3). Drains were sampled and disinfected once a day for 5 days (d1-5) and were also sampled at week 1, 2, 3 and 9 after the last disinfection (w1, w2, w3, w9). The boiling water disinfection was carried out on two sink drains in patient rooms (light green bars) and on one handwashing station outside patient rooms (dark green bars). CFU = Colony-forming units. Missing data is represented by “X”.

To further investigate the efficacy of thermal disinfection, four methods were then compared in multiple sinks. The boiling water disinfection was carried out on two sink drains in patient rooms and on one handwashing station outside patient rooms. It is important to note that the two patient room drains had been previously disinfected weekly with boiling water for seven weeks (Fig 1, sampling 2), about eight weeks before the start of this comparison (Fig 4). Thus, these drains exhibited significantly (Mann-Whitney U test, p < 0.05) lower levels of HPC and ICC concentrations before the onset of treatment (d1) than all the other drains, showing the long-term effect of boiling water treatment on bacterial loads in drains. Despite having a substantial and well-established biofilm, a decrease in bacterial concentrations was observed in the hand-washing station during the boiling water disinfection process.

Except for the two patients room drains disinfected with boiling water, thermal disinfection treatments significantly reduced (Wilcoxon signed-rank test, p < 0.01) both HPC and ICC concentrations in the drains (Fig 4). Mean reductions for HPC and ICC were respectively 6-log and 1.9-log for hot tap water flushing at 58–59°C, 4.5-log and 1.2-log for hot tap water flushing at 47–48°C, 5.4-log and 2.8-log for steam disinfection and 4.9-log and 2.1-log for the boiling disinfection on the handwashing station. However, after treatment was discontinued, HPC levels increased progressively to reach pre-treatment levels after 9 weeks. The impact on ICCs was more progressive and persistent even 9 weeks after the end of disinfection treatments. Unsurprisingly, hot water flushing at higher temperatures (58°C and 59°C) was more efficient than flushing at lower temperatures (47°C and 48°C).

### 3.2 Effect of disinfection treatments on three opportunistic pathogens

**Fig 5:**
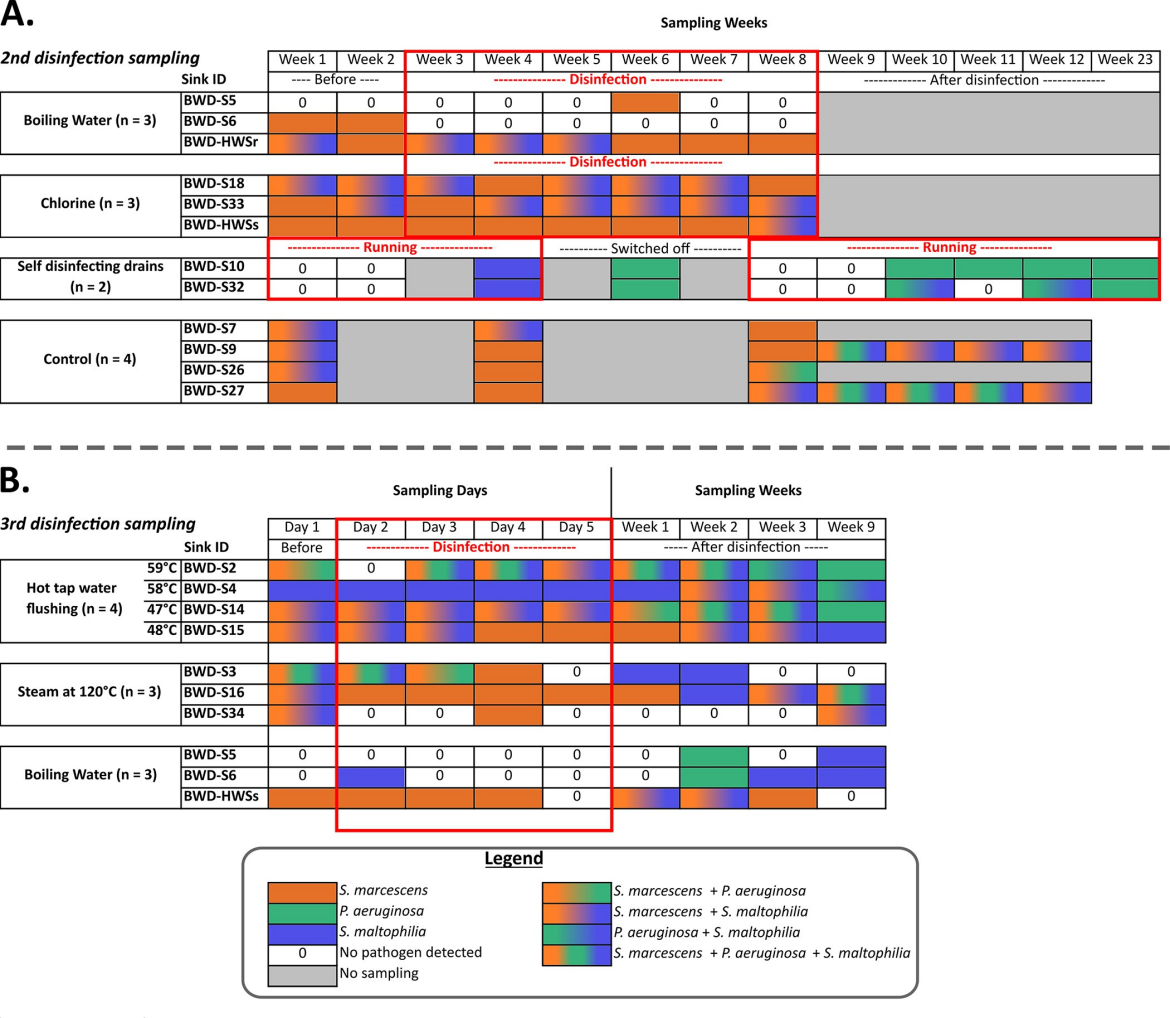
PCR detection results of three opportunistic pathogens (OPs) in drains using HiSST schemes for various disinfection methods. Table (A) shows OP detection for three specific treatments (boiling water, chlorination, self-disinfecting drains) and the no treatment control. Table (B) presents the OP detection for three thermal treatments (30-minute hot tap water flushing, steam at 120°C, boiling water). Each cell in the table represents a sink drain sample, with colors (orange, blue, and green) indicating the presence of *S*. *marcescens*, *S*. *maltophilia*, and *P*. *aeruginosa*, respectively. Sinks in NICU patient rooms are identified by the letter "S" followed by a number. The sampling day (3-digit 365-day calendar) is identified in the third row of the tables, preceded by ’1’ for 2021 or ’2’ for 2022). Acronyms: Biofilm Water Drain (BWD), Hand Washing Station in intensive care unit (HWSs), Hand Washing Station in intermediate care unit (HWSr).

The effectiveness of the disinfection methods in reducing the presence of selected OPs (*S*. *marcescens*, *S*. *maltophilia*, and *P*. *aeruginosa*) in sink drains was also assessed (Fig 5). Fig 5 shows the results of OPs prevalence for self-disinfecting commercial drains (n = 2), chlorine treatment (n = 3), boiling water treatment (n = 3), 120°C steam treatment (n = 3), hot tap water flushing (n = 4), and untreated controls (n = 4). Given the high positivity of sink drains for *S*. *marcescens* and the recurring colonization episodes by this OP [51], further in-depth investigations were undertaken to investigate its presence in sink drains via genotyping analysis. Hence, *S*. *marcescens* positive samples were analyzed using the HiSST method to assess the presence of specific strains before, during, and after treatment. Genotyping results for *S*. *marcescens* positive samples confirmed the persistence of unique strains in both control and treated sinks. Additionally, analysis of HiSST profiles enhanced the resolution of our observations by verifying the ability of genotypes to recolonize drains.

**Fig 6:**
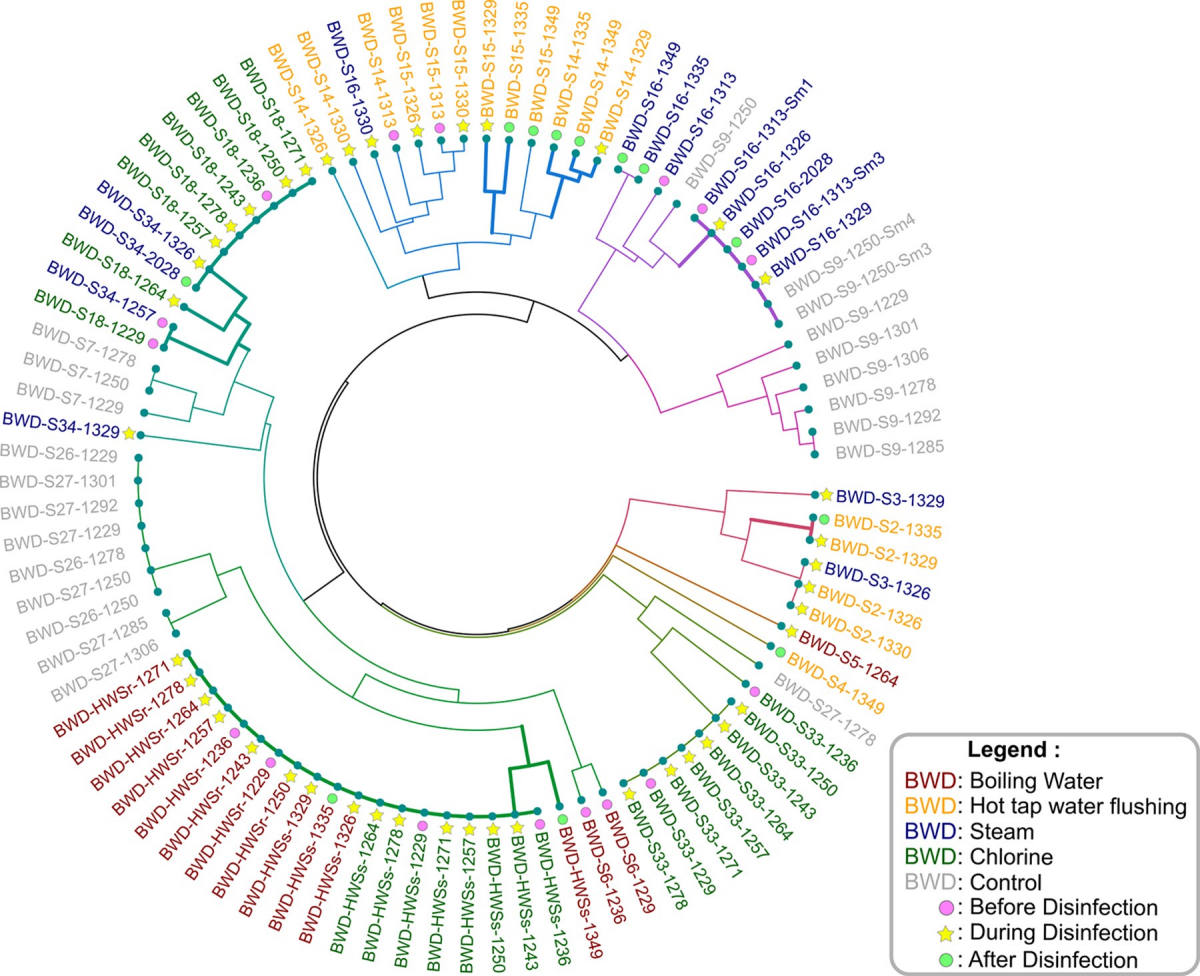
Genotype comparison of *S*. *marcescens*-positive sink drain samples collected before, during and after five treatments. This circular UPGMA dendrogram was based on Jaccard distance, calculated from HiSST profiles of *gabR*, *bssA* and *dhaM* loci among positive sink drain samples. Samples were obtained during drain disinfection campaigns conducted in the NICU between 2021 and 2022. Samples with a similar HiSST profile are more likely to be colonized by the same strain. Branches with identical or similar genotypes detected before/during and after treatments in a same sink drain are highlighted by thicker lines. Samples are named as follows: sample type (BWD for biofilm and drain water combined)—Sink ID–Sampling day (preceded by ’1’ for 2021 or ’2’ for 2022). Isolates are identified by "Sm#" at the end of the sample name. Sinks in NICU patient rooms are identified by the letter "S" followed by a number. Acronyms: Hand Washing Station in intensive unit (HWSs), Hand Washing Station in intermediate unit (HWSr).

Chlorine treatments were found to be ineffective in reducing ICCs and eliminating positivity for OPs. Interestingly, identical genotypic profiles for *S*. *marcescens* were detected before and during treatment (Fig 6, samples in green). This observation indicates that chlorine failed to reduce drain colonization by *S*. *marcescens*.

Similarly, flushing with hot tap water did not reduce positivity for the three studied OPs (Fig 5B); however, higher hot water temperatures led to a greater decrease in ICCs. While this treatment led to a slight reduction in HPCs (Fig 3), genetic profiles of *S*. *marcescens* remained unchanged before, during, and after flushing, indicating their persistence in the drains despite the intervention (Fig 6).

On the other hand, boiling water treatment lowered OP positivity, even when excluding sinks that were already negative before the treatment. A sink colonized by *S*. *marcescens* was effectively disinfected upon the first application of boiling water treatment and remained negative for this species throughout the study period (Fig 5B). However, ICCs bounced back after the end of treatment and sporadic contamination by *P*. *aeruginosa* was observed after treatment cessation. Furthermore, while *S*. *maltophilia* managed to recolonize sink drains S5 and S6 three weeks after the end of treatment, it appears that boiling water treatment prevented a sustainable recolonization of *S*. *marcescens* and *P*. *aeruginosa* in sink drains (negative at the ninth sampling week). Regarding sink HWSr (handwashing station in the intermediate care unit), disinfection with boiling water failed to eliminate *S*. *marcescens* but proved effective against *S*. *maltophilia* from the third week of treatment. This sink is similar to the HWSs (handwashing station in the intensive care unit), which also exhibits high bacterial loads with a substantial presence of biofilms, potentially explaining the resistance of *S*. *marcescens* to thermal treatment. The sink HWSs’ drain was positive for *S*. *marcescens* after disinfection with chlorine during the second campaign (Fig 5A). However, the HWSs drain was negative for *S*. *marcescens* on the fifth day of boiling water disinfection (Fig 5B). Despite this intervention, the genotype identified before the treatment recolonized the same drain after the first week following the end of treatment, and persisted until the third week after disinfection (Fig 6, samples in red). After this period, this drain tested negative for *S*. *marcescens* at week 9.

Results for the three sinks subjected to steam disinfection at 120°C suggested effectiveness in eliminating OPs from sinks S3 and S34, with no OPs detected for several weeks post-treatment (sink S34).

Self-disinfecting drains were not positive for OPs at the onset of testing, limiting our ability to evaluate their ability to reduce OP positivity. Nevertheless, the results reveal a reduction in HPCs and ICCs by 7 and 4 log, respectively, and suggest that self-disinfecting drains may effectively prevent OP colonization. A week before switching off the self-disinfecting drains (week 4), both sample sink drains were positive for *S*. *maltophilia*. Then, during the three weeks where the self-disinfecting drains were unplugged (weeks 5, 6, 7), both drains became positive for *P*. *aeruginosa* on the same sampling date (week 6). Three weeks after restarting the self-disinfecting drains, *P*. *aeruginosa* was once again detected, suggesting its ability to persist once reseeded, despite resuming the operation of the self-disinfecting drain operation.

A most important finding of this study is the rapid recolonization of drains by OPs, ICCs and HPCs following the end of disinfection treatment, regardless of the type of treatment applied. For drains treated with boiling water, *P*. *aeruginosa* was detected in two sinks, two weeks after the end of treatment, and *S*. *maltophilia* was detected in all three sinks tested. Certain *S*. *marcescens* strains recolonized drains within one to four weeks after steam or boiling water treatment (Fig 6, sample BWD-S34-2028 in blue for steam disinfection, and sample BWD-HWSs-1335 in red for boiling water disinfection). However, hot tap water flushing treatment does not appear to prevent *P*. *aeruginosa* drain recolonization immediately after the end of treatment. Three of four sinks tested positive for *P*. *aeruginosa* after four weeks, whereas only one was positive before the treatment. In contrast, *S*. *marcescens* seems to exhibit more sustained vulnerability to hot and boiling water treatments. Indeed, nine weeks after discontinuing these two treatments, *S*. *marcescens* was no longer detected in sink drains. Nevertheless, several HiSST genotypes were identified a few weeks after the disinfection process ─ up to three weeks later ─ in four sink drains (#S2, #S14, and #S15, following hot tap water flushing, and in the HWSs after boiling water treatment). These sinks had been previously colonized by the same HiSST genotypes during or before the treatment (orange and red samples in Fig 6).

## 4. Discussion

### 4.1 Advantages and limitations of various disinfection methods

Based on this study’s findings and our experience, Table 1 summarizes the characteristics and efficacy of the different methods tested, and identifies their main advantages and limitations. The selection of a disinfection method should be primarily driven by considerations of effectiveness in reducing the target OPs. However other considerations such as the feasibility of implementation, costs, potential impacts on plumbing materials, risks of scalding and chemical/microbiological exposure, and site-specific constraints may influence this selection. The relevance of these considerations may differ across various facilities.

**Table 1:** Main characteristics, advantages and limitations of drain disinfection methods.

Disinfection methods	Estimated efficacy ^1^			Sink downtime	Advantages	Limitations
	HPC	ICC	OP presence			
*Shock chlorination—discontinuous*						
Chlorine (20 mg/L, 30 min with valve)	+	─	─	30 min	• Easy to implement • No need for technical expertise • Low cost	• Chemical disinfection • Requires valve installation • Routine manual intervention • Harmful to water treatment plants • Degassing control needed • Risk of degradation of metallic and elastomer/polymer-based materials
*Physical disinfection—discontinuous*						
Boiling water (30 min with valve)	+++	++	+++	30 min	• Easy to implement • No need for technical expertise • Low cost	• Requires valve installation • Routine manual intervention • Risk of degradation of elastomer/polymer-based materials (pipes and gaskets) at 100°C
Hot water flushing (30 min)	+++	++	─	30 min	• Easy to implement • No need for technical expertise • No installation required • Low cost	• Routine manual intervention • High water consumption • Aerosol control needed
Steam (120°C, 3 min)	+++	++	++	10–15 min	• Physical disinfection combination (high pressures and temperatures)	• Routine manual intervention • Need for high technical expertise • Expensive, bulky and noisy device • High risk of degradation of elastomer/polymer seals and PVC pipes at high pressure and 120°C • Aerosol and steam control needed
*Physical disinfection—continuous*						
Self-disinfection drains	+++	++	+++	None	• Physical disinfection combination (high temperature and vibration) • Self-disinfection	• Expensive device • Requires drain replacement • Requires power supply • Vulnerability to interruption

^1^ The effectiveness of each disinfection method was extrapolated from observations of this study and determined using the following scoring criteria: ─: None; +: low; ++: moderate; +++: good. HPC = Heterotrophic Plate Count; ICC = Intact Cell Count; OP = Opportunistic Pathogen.

In this study, bacterial disinfection indicators included total and viable bacteria by flow cytometry, culturable bacteria and positivity of OPs by PCR. Employing molecular methods to track the presence of OP was important for ensuring a rigorous assessment of treatment effectiveness, as discussed in the limitations section. As expected, for bacterial loads, ICCs measured by flow cytometry were systematically higher than HPCs measured with traditional culture-based methods. More importantly, responses to disinfection varied widely depending on the indicator considered. The observed variation can be explained by the ability of bacteria to enter a viable but nonculturable state when exposed to environmental stresses or integrated into biofilms. This renders them challenging to cultivate, while remaining detectable through the use of flow cytometry [56]. Additionally, it highlights the limitations associated with conventional culture-based methods, such as HPCs, for assessing the effectiveness of disinfection procedures. However, biofilm presence notably impacts disinfection method efficacy, highlighting its critical consideration in protocol selection.

High dosage chlorine disinfection, even when optimized by the use of a valve to maximize contact time, was notably ineffective in reducing the concentration of viable bacteria, as indicated by persistent high concentrations of HPCs and ICCs. Furthermore, it had no impact on the positivity of the three OPs studied. Other studies show the poor performance of sodium hypochlorite disinfection methods in sink drains [32, 57, 58]. While chlorine is effective against various bacteria, its use may result in the emergence of resistant strains like *P*. *aeruginosa* [59]. Repeated shock chlorination poses risks such as the degradation of metallic piping and fittings, along with corrosion of elastomers and plastic piping, seals, and gaskets commonly used in building water systems.

The most effective disinfection method was boiling water, enhanced by the use of a valve, resulting in a reduction in HPCs, ICCs, and the presence of OPs. Implementation involves installing a valve and isolating the P-Trap with a sleeve to maintain high temperatures during the treatment process. Repeated treatment for several weeks resulted in an observable upward trend in ICCs, suggesting a potential gradual adaptation within the microbial population. While effective, the repeated use of boiling water for disinfection may compromise the integrity of pipe joints over time. Its applicability should consider the materials present in the drainage system. Future studies should also explore disinfection with hot water between 60°C and 90°C, using the same protocol as boiling water, to enhance feasibility and minimize the risk of burn to the personnel.

Flushing with hot tap water (47–59°C) is a simple disinfection method, but it results in significant water wastage during the 30-minute usage. This disinfection treatment has demonstrated higher effectiveness than chlorine treatments in reducing HPCs and ICCs. However, hot tap water flushing was ineffective in eliminating OP positivity in drains. The three OPs studied can resist thermal disinfection at temperatures below 60°C, consistent with the literature [60–62]. During the flushing process, temperature measurements at the tap water outlets ranged from 47°C to 59°C, but notably decreased when flowing through the drainpipe. These temperatures are likely not sufficient to effectively eliminate OPs in drains, especially within biofilms, which can be formed independently by *P*. *aeruginosa*, *S*. *marcescens* or *S*. *maltophilia* [63–66]. Indeed, biofilms can serve as protective barriers against harsh conditions [28], including hot temperatures (around 45°C) [67], making it challenging to eradicate well-established OPs. Since PCR-based positivity detection cannot be indicative of OP concentrations, the reduction in both culturable bacteria and ICCs could be associated with a decrease in pathogen concentration. However, the persistent presence of OPs in drains, even at low levels, along with the simultaneous reduction in culturable bacteria and ICCs, could contribute to a rapid drain recolonization by these pathogens. Indeed, reduced competition can create a favorable environment for OPs to thrive, such as *P*. *aeruginosa*. Temperatures of 40°C or higher can also play a significant role in the selection of more virulent bacteria in environmental reservoirs [68].

Steam treatment was also highly effective in reducing concentrations of ICCs and HPCs, and very effective in lowering OP positivity. Steam application is a rapid process, albeit requiring the handling of bulky devices, that can be easily performed by system operators. However, repeated steam treatments cannot be conducted in most plastic piping and are most likely to damage elastomers present in seals and pipe joints.

Considering both effectiveness and impact, boiling water and steam treatments show the most promising results against OPs, as the temperature applied is high enough to reduce positivity and decrease biofilm viability [69]. Nevertheless, their effectiveness appears to be limited in some drains where bacterial biofilm is abundant and well-developed, necessitating pre-treatment against biofilms to improve the effectiveness of heat treatments. However, the handwashing station at the NICU entrance (i.e., HWSs), which initially had a high bacteria concentration, showed a negative result for *S*. *marcescens* after only five days of boiling water disinfection (Fig 5) and again after the ninth week following the completion of disinfection. During the three weeks after the disinfection process, the drain tested positive for *S*. *marcescens*. The absence of *S*. *marcescens* detection on certain dates may be due to a reduction in population concentration, close to the detection limit and, depending on the specific sampling zone, below the detection limit. Another explanation could be attributed to the treatment effectiveness in decontaminating the sampled section (from the P-Trap to the sink strainer) without decontaminating other downstream parts of the water drainage system, situated beyond the trap valve, unaffected by the decontamination process. *S*. *marcescens* might have survived within the water drainage system, enabling it to recolonize the sampled drain. Nevertheless, the capacity of this species to persist could be limited after the thermal shock treatment if the population density falls below their threshold for viability, as previously noted by Ricker et al. in their study *on Pseudomonas aeruginosa* biofilms [69]. Additionally, as culturable bacteria returned to their initial levels, the microbiome may have limited the ecological niche of *S*. *marcescens*. This limitation could be attributed to a reduction in its "ecological space" or to the principle of competitive exclusion. These explanations could partly elucidate its absence during the sampling conducted in the ninth week, specifically within the sampled areas of the drain on that particular day.

Although efficacy could not be fully established for self-disinfecting drains due to the limited number of available test drain devices and the absence of reference levels before their installation, results strongly suggest that these devices effectively prevented pathogen establishment when functioning properly. These observations align with prior reports, suggesting that self-disinfection devices can reduce HAI [18, 27], and indicating a decrease in the prevalence of *Klebsiella pneumoniae* carbapenemase–producing organisms after the implementation of such sink trap devices in an ICU [47]. However, periods of device shutdown may occur as a result of power failures, system faults, or inadvertent unplugging. The rapid and persistent colonization of these drains by *P*. *aeruginosa*, despite resuming self-disinfection, highlights this device limitations, diverging from conclusions drawn in a previous study [27]. In such cases, an intensive pre-disinfection procedure, using a combined decontamination approach [70], should be considered before restarting the device. Further research is necessary to validate their long-term efficacy and evaluate the risk of recolonization in case of unplanned interruption, particularly considering their higher cost. Indeed, the cost for one device is approximately 3,500$CAD, excluding professional intervention costs for drain replacement.

### 4.2. Application of the actionable evidence to infection prevention

Numerous studies have discussed the significant influence of sink design on the prevalence and dispersion of contaminated droplets and aerosols in the patient environment [3, 16, 17, 23, 25, 58, 71, 72]. In the studied NICU, sinks are already well designed with key protective features including drains that are not directly aligned with the water jet, positioned away from the patient’s bed (approximately 2–3 meters), and equipped with recent deep drains for efficient drainage. Nevertheless, clinical evidence, environmental drain monitoring and disinfection interventions in this study reveal the importance of drain contamination. Indeed, in response to multiple episodes of *S*. *marcescens* colonization or infection in patients between 2019 and 2021 [51], a targeted approach was implemented to disinfect problematic sinks located in rooms occupied by positive patients. After the third sampling campaign, all NICU drains (n = 35) were steam disinfected and, almost half (n = 15) were also randomly selected to be flushed with hot tap water once a week for four consecutive weeks. Two months later, all 45 sink drains in intermediate care rooms were steam disinfected. After the implementation of these new procedures for thermal disinfection of drains, the number of cases of nosocomial *S*. *marcescens* colonization decreased in the investigated NICU in 2022. During the 2021 outbreak, 22 cases were reported, compared to 9 cases in 2022 after implementation of drain disinfection. Despite these measures, new positive cases of *S*. *marcescens* colonization were recorded in 2023. However, sink drains were not sampled in 2022 and 2023. Thus, it remains uncertain whether clinical strains came from the drains, parents, or patient-to-patient transmission. These observations underline the limitations of current disinfection method to minimize the presence of OPs in drains and reduce the risk of HAI.

### 4.3. Limitations in assessing the efficacy of treatments against opportunistic pathogens

The presence of relic DNA can lead to false-positive results when detecting OPs [73–75]. This risk can be real in sink drains, since stagnant water in siphons can retain extracellular DNA from dead cells. However, this bias remains limited, as our samples were taken once a week or every other week. Additionally, most sinks in the study were regularly used, ensuring the consistent flushing of pipes. Furthermore, during disinfection tests, the studied OPs were not quantified, even though certain treatments might have effectively reduced their concentrations. When combined with the challenges related to relic DNA, our methodology proved to be quite stringent in estimating treatment effectiveness. This stringency could explain the limited number of conclusive results obtained. Another limitation is inherent to the unbalanced experimental design for treatment types. Also, the number of biological replicates for each test was low, due to the reality of the field (e.g., difficulty in performing steam disinfection in the presence of patients, lack of equipment or sinks available for testing). It should be noted that these tests were carried out in a context of *S*. *marcescens* case clusters, requiring rapid intervention in the NICU. Despite these limitations, our findings emphasize the inadequacy of chlorine treatments and hot water flushing in the tested conditions. They also point to the potential of high-temperature disinfection (>80°C) as a promising treatment approach if materials allow it. Nevertheless, long-term treatment efficacy should be validated on a broader scale, with controlled conditions and across different NICUs.

## 5. Conclusion

Results from this study provide actionable information to assist infection control teams in defining drain disinfection programs to limit the risks associated with OPs in drains. The findings suggest a potential efficacy of boiling water and steam disinfection against bacterial indicators and OPs presence in drains. These results will guide future investigations aimed at proving the efficacy of high temperature treatments in drains, involving a larger number of sinks and various hospital establishments. In contrast, results highlight the ineffectiveness of chlorine disinfection and hot water flushing in removing OPs from drains. Currently, high-temperature disinfection stands out as a potential, albeit temporary, solution to reduce sink contamination during an ongoing epidemic.

This study underscores the persistent challenge of eradicating OPs in sink drains despite rigorous disinfection efforts. A most important finding of this study is the rapid recolonization of drains by OPs, ICCs and HPCs following the end of disinfection treatment, regardless of the type of treatment applied. Thus, the combination of technical measures and preventive protocols for healthcare workers is important to prevent contamination of patients by sink-colonizing bacteria, given the challenge of systematic recolonization events. A critical component of chemical and physical intervention is the duration of treatment that defines the intensity of the exposure. To ensure sufficient contact time, valves should be installed after the P-Traps. Other approaches should be explored, such as a combined strategy involving the destabilization of biofilms through an initial mechanical or biological pre-treatment of drains (e.g., utilizing digestive enzymes to weaken extracellular polymeric substances in biofilms), followed by thermal shock (e.g., boiling water). Although there remain unresolved questions, our findings offer some practical solutions to manage OP sink drain colonization, and contribute to defining a comprehensive set of solutions for the various situations in hospitals.

Finally, in order to achieve more efficient disinfection of drains and prevent infection, there is an urgent need for an enhanced understanding of pathogen ecology within NICUs. As demonstrated in our previous study, sinks are significant reservoirs of OPs and may contribute to HAIs, but the primary sources of HAIs often involve horizontal transmission of OPs [51]. While we await more comprehensive, evidence-based solutions validated by larger studies across various ICUs, the focus should be on controlling infection vectors. Sinks should be kept away from patients to minimize the dissemination of contaminated droplets and aerosols. Given the challenges of implementing effective P-Trap disinfection regimes, additional physical barriers such as the use of disposable drain covers, could also be considered [76].

## Supporting information

S1 Fig Illustrations of the treatment systems used in this study. (PDF)

S2 Fig Flow cytometry total cell count (TCC) concentrations in drains before and after the installation of a self-disinfecting drain and a new P-Trap. (PDF)

S3 Fig Concentrations of (A) heterotrophic plate count (HPC), (B) flow cytometry intact cell count (ICC) and (C) flow cytometry total cell count (TCC) in self-disinfecting drains and control drains.
(PDF)

S4 Fig Concentrations of total cell count (TCC) in drains before, during and after chlorine and boiling water disinfection. (PDF)

S5 Fig Concentrations of flow cytometry total cell count (TCC) in drains during and after thermal disinfection. (PDF)

S6 Fig Chronology of nosocomial *S*. *marcescens* colonizations and infections from 2019 to 2023 in the NICU. (PDF)

S1 Table Detailed sequencing information including barcodes, read proportions, and ASV counts. (XLSX)
