# Supplementary material for: Disinfection of sink drains to reduce a source of three opportunistic pathogens, during Serratia marcescens clusters in a neonatal intensive care unit
Source: PLoS One. 2024 Jun 12;19(6):e0304378. doi: 10.1371/journal.pone.0304378 (PMC11168660; doi:10.1371/journal.pone.0304378)
Supplement: S1 Fig — (PDF) [file pone.0304378.s001.pdf]

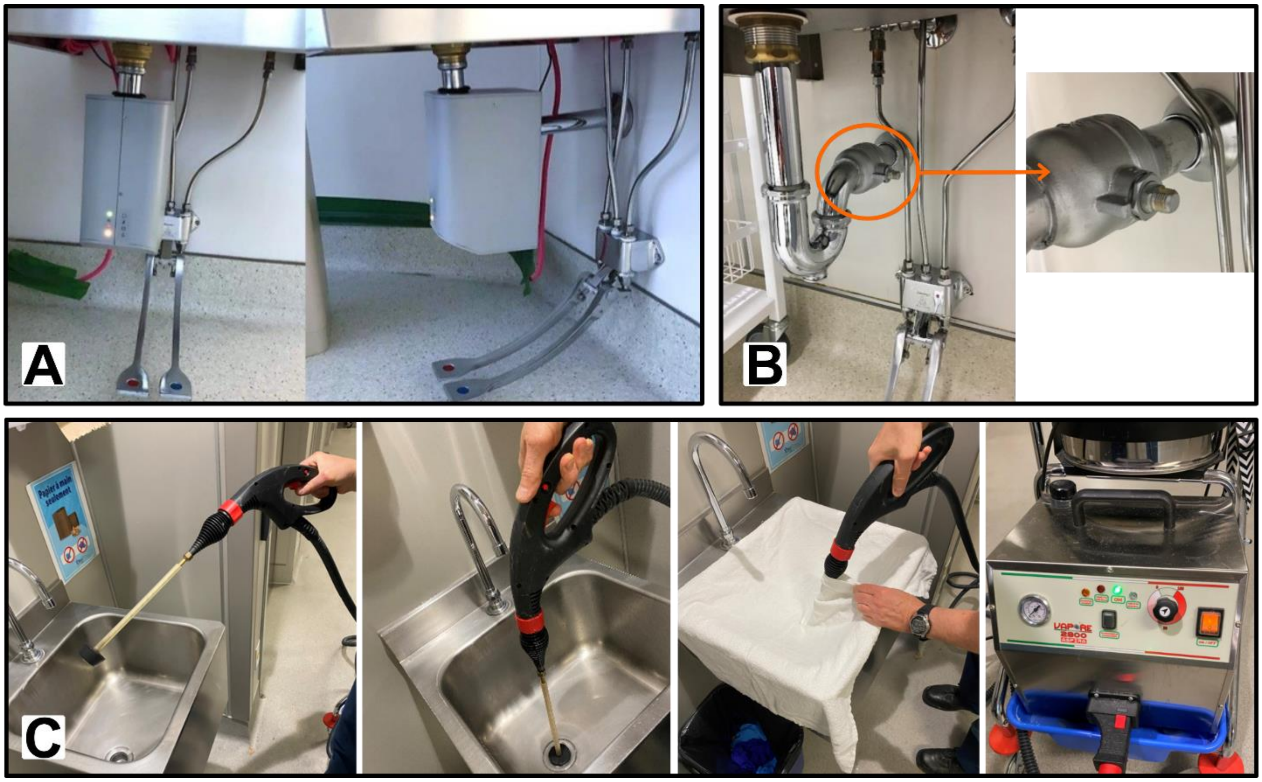

Figure S1. Illustrations of the treatment systems used in this study.

In the first panel (A), the self-disinfection device (KLEANIK™ Sink Disinfection System, Surgmed Group, Montreal, QC, Canada) is depicted, installed on a sink drain. Stainless steel sinks are equipped with two pedals to activate hot and cold water at the faucet. Panel (B) shows a valve installed on the drain between the P-Trap outlet and the wall, used during the boiling water and chlorine treatment process. In panel (C), the steam disinfection system is shown in four images: the first image (left image) shows the hose of the steam disinfection system; the second (middle-left image) shows it placed inside the drain and securely fastened to the strainer with a rubber stopper; the third (middle-right image) shows a towel placed over the sink during the disinfection process; and the fourth (right image) shows its connection to the steam generator.
