# Supplementary material for: Disinfection of sink drains to reduce a source of three opportunistic pathogens, during Serratia marcescens clusters in a neonatal intensive care unit
Source: PLoS One. 2024 Jun 12;19(6):e0304378. doi: 10.1371/journal.pone.0304378 (PMC11168660; doi:10.1371/journal.pone.0304378)
Supplement: S2 Fig — (PDF) [file pone.0304378.s002.pdf]

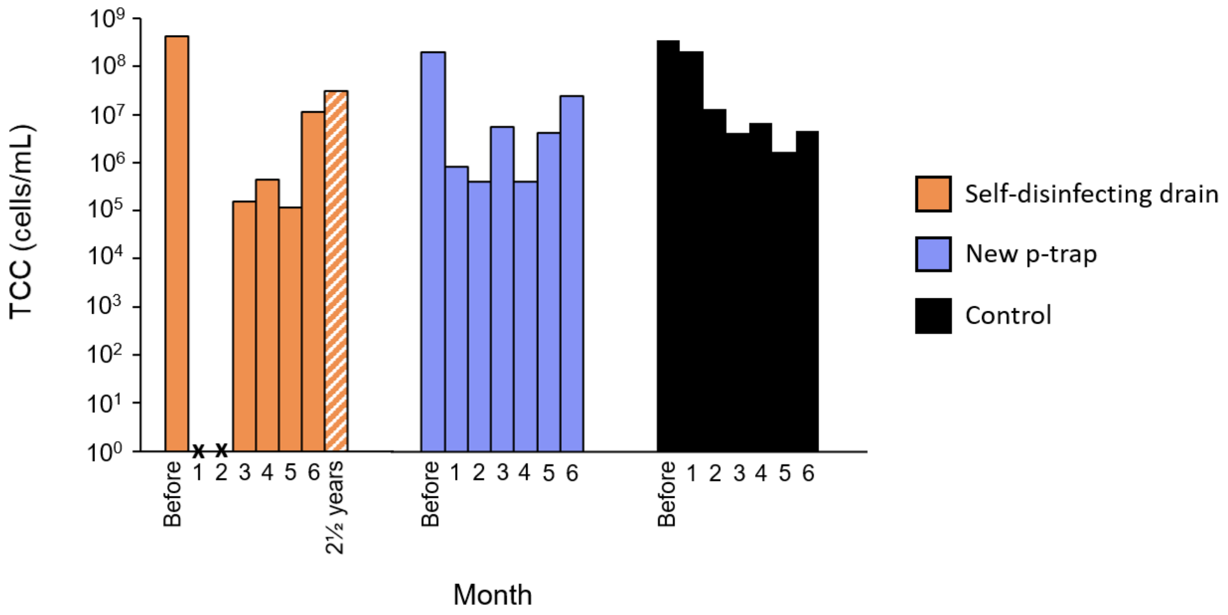


**Figure S2. Flow cytometry total cell count (TCC) concentrations in drains before and after the installation of a self-disinfecting drain and a new P-Trap.**

Drains were sampled once a month for six months. Bars represent the mean of replicates for each sample. Missing data is represented by "X".
