# Supplementary material for: Disinfection of sink drains to reduce a source of three opportunistic pathogens, during Serratia marcescens clusters in a neonatal intensive care unit
Source: PLoS One. 2024 Jun 12;19(6):e0304378. doi: 10.1371/journal.pone.0304378 (PMC11168660; doi:10.1371/journal.pone.0304378)
Supplement: S3 Fig — Concentrations of (A) heterotrophic plate count (HPC), (B) flow cytometry intact cell count (ICC) and (C) flow cytometry total cell count (TCC) in self-disinfecting drains and control drains. (PDF) [file pone.0304378.s003.pdf]

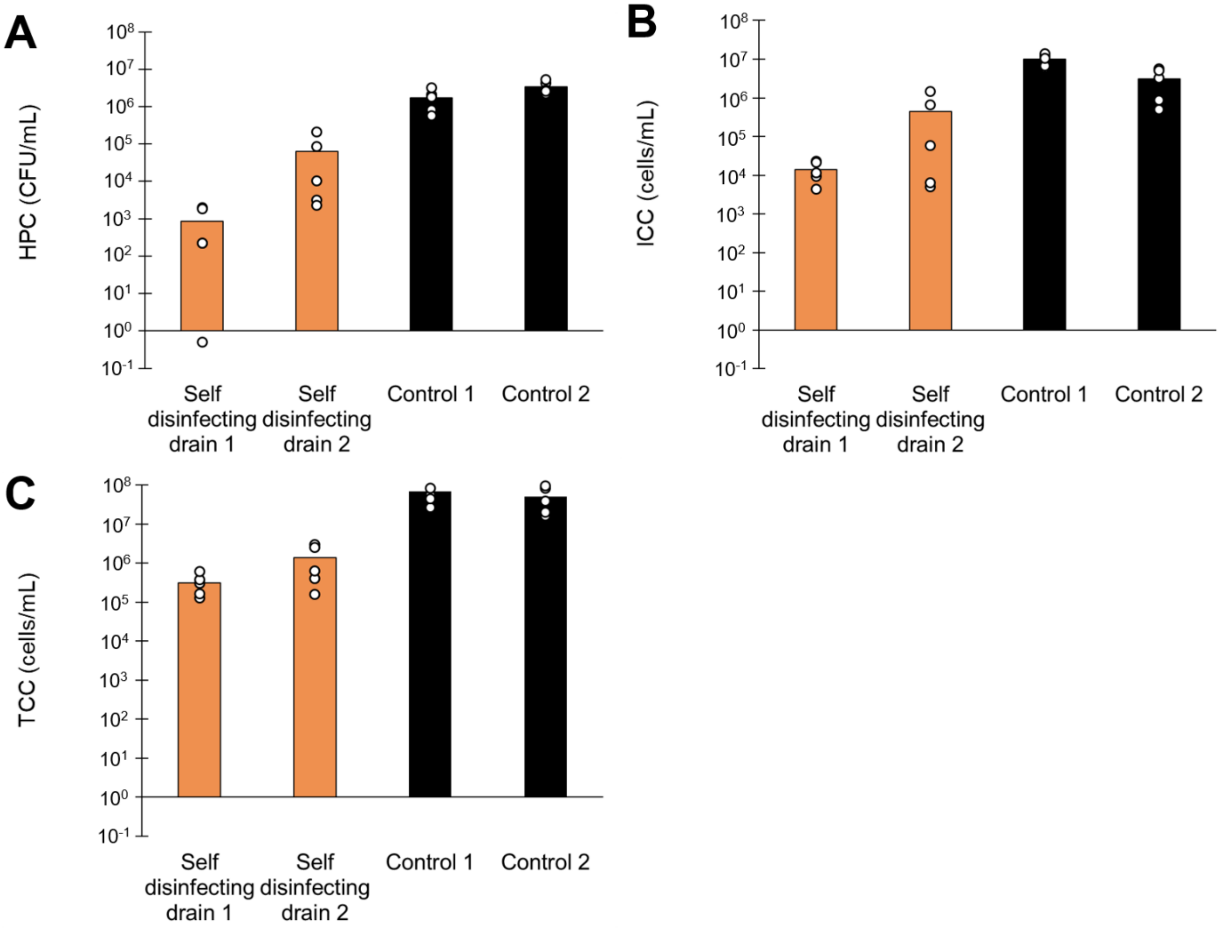

Figure S3. Concentrations of (A) heterotrophic plate count (HPC), (B) flow cytometry intact cell count (ICC) and (C) flow cytometry total cell count (TCC) in self-disinfecting drains and control drains.

Drains were sampled once a week for 5 weeks. Bars represent the mean of the replicates for each sample (symbolized by circles) of each drain. CFU = Colony-forming units.
