# Supplementary material for: Disinfection of sink drains to reduce a source of three opportunistic pathogens, during Serratia marcescens clusters in a neonatal intensive care unit
Source: PLoS One. 2024 Jun 12;19(6):e0304378. doi: 10.1371/journal.pone.0304378 (PMC11168660; doi:10.1371/journal.pone.0304378)
Supplement: S4 Fig — (PDF) [file pone.0304378.s004.pdf]

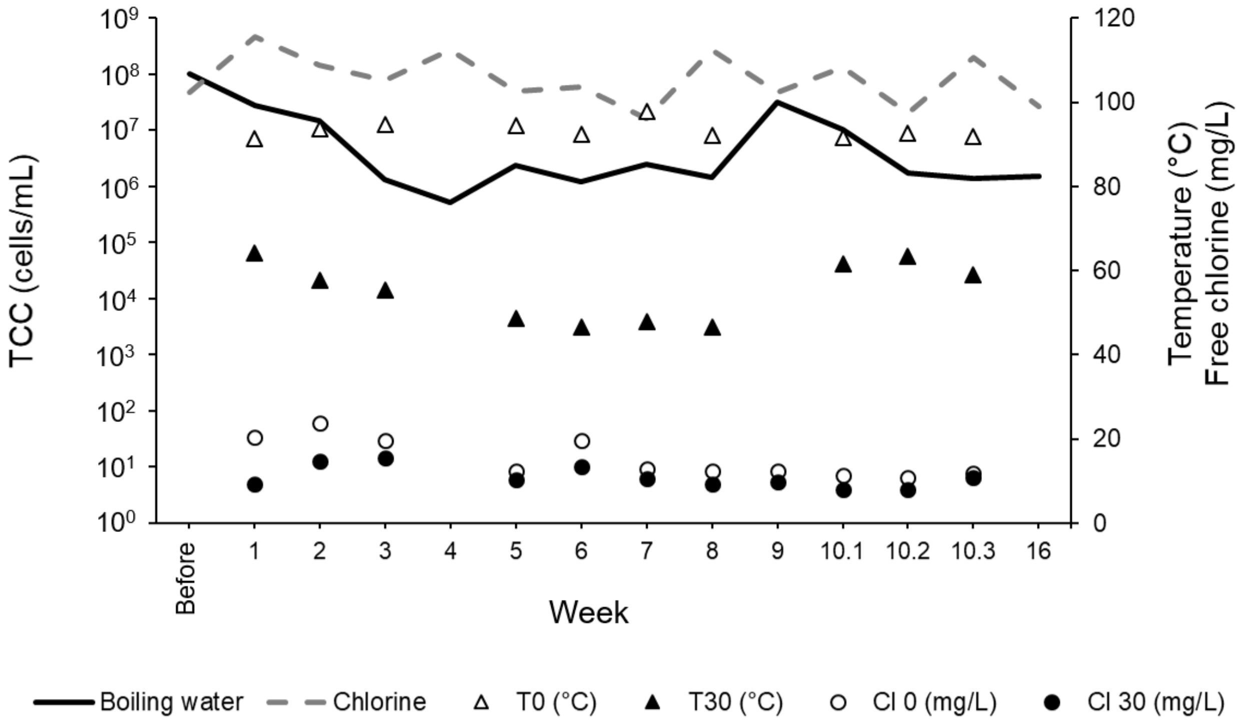

Figure S4. Concentrations of total cell count (TCC) in drains before, during and after chlorine and boiling water disinfection.

Boiling water results are full line and chlorine results are dotted line. Initial (T0, unfilled triangles) and final (T30, filled triangles) temperatures were measured for the boiling water disinfection as well as the initial (Cl 0, unfilled circles) and final (Cl 30, filled circles) free chlorine for the chlorine disinfection. CFU = Colony-forming units.
