# Supplementary material for: Disinfection of sink drains to reduce a source of three opportunistic pathogens, during Serratia marcescens clusters in a neonatal intensive care unit
Source: PLoS One. 2024 Jun 12;19(6):e0304378. doi: 10.1371/journal.pone.0304378 (PMC11168660; doi:10.1371/journal.pone.0304378)
Supplement: S5 Fig — (PDF) [file pone.0304378.s005.pdf]

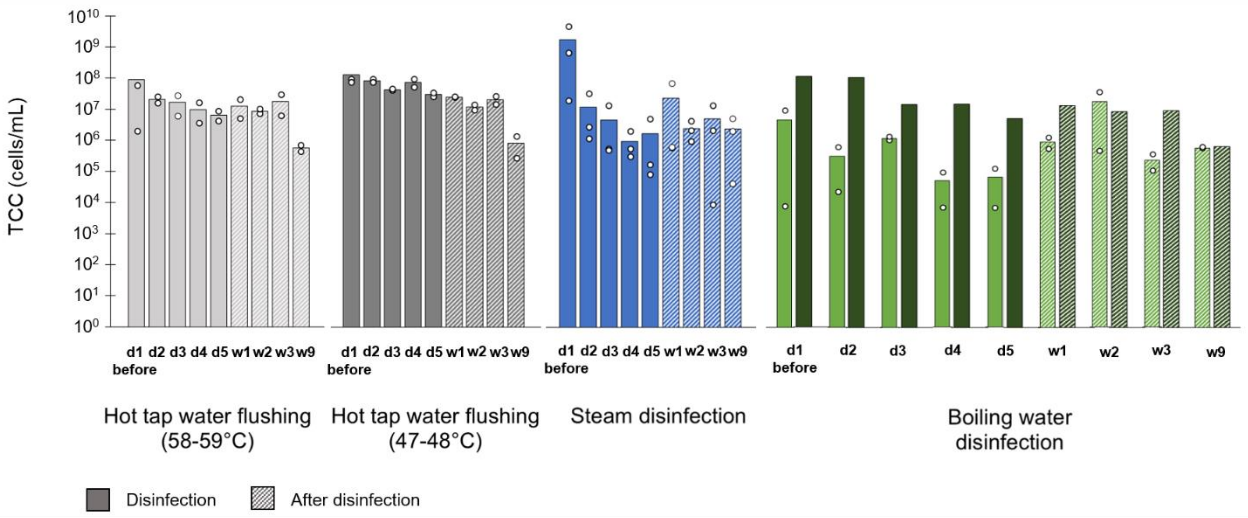

Figure S5. Concentrations of flow cytometry total cell count (TCC) in drains during and after thermal disinfection.

Bars represent the mean of replicates for each sample (symbolized by empty circle) from sinks with hot tap water flushing (n = 2 at 58-59 °C; and n = 2 at 47-48°C), steam disinfection (n = 3) and boiling water disinfection (n = 3). Drains were sampled and disinfected once a day for 5 days (d1-5) and were also sampled at week 1, 2, 3 and 9 after the last disinfection (w1, w2, w3, w9). The boiling water disinfection was carried out on two sink drains in patient rooms (light green bars) and on one handwashing station outside patient rooms (dark green bars). Colony-forming units = CFU. Missing data is represented by “X”.
