# Supplementary material for: Disinfection of sink drains to reduce a source of three opportunistic pathogens, during Serratia marcescens clusters in a neonatal intensive care unit
Source: PLoS One. 2024 Jun 12;19(6):e0304378. doi: 10.1371/journal.pone.0304378 (PMC11168660; doi:10.1371/journal.pone.0304378)
Supplement: S6 Fig — (PDF) [file pone.0304378.s006.pdf]

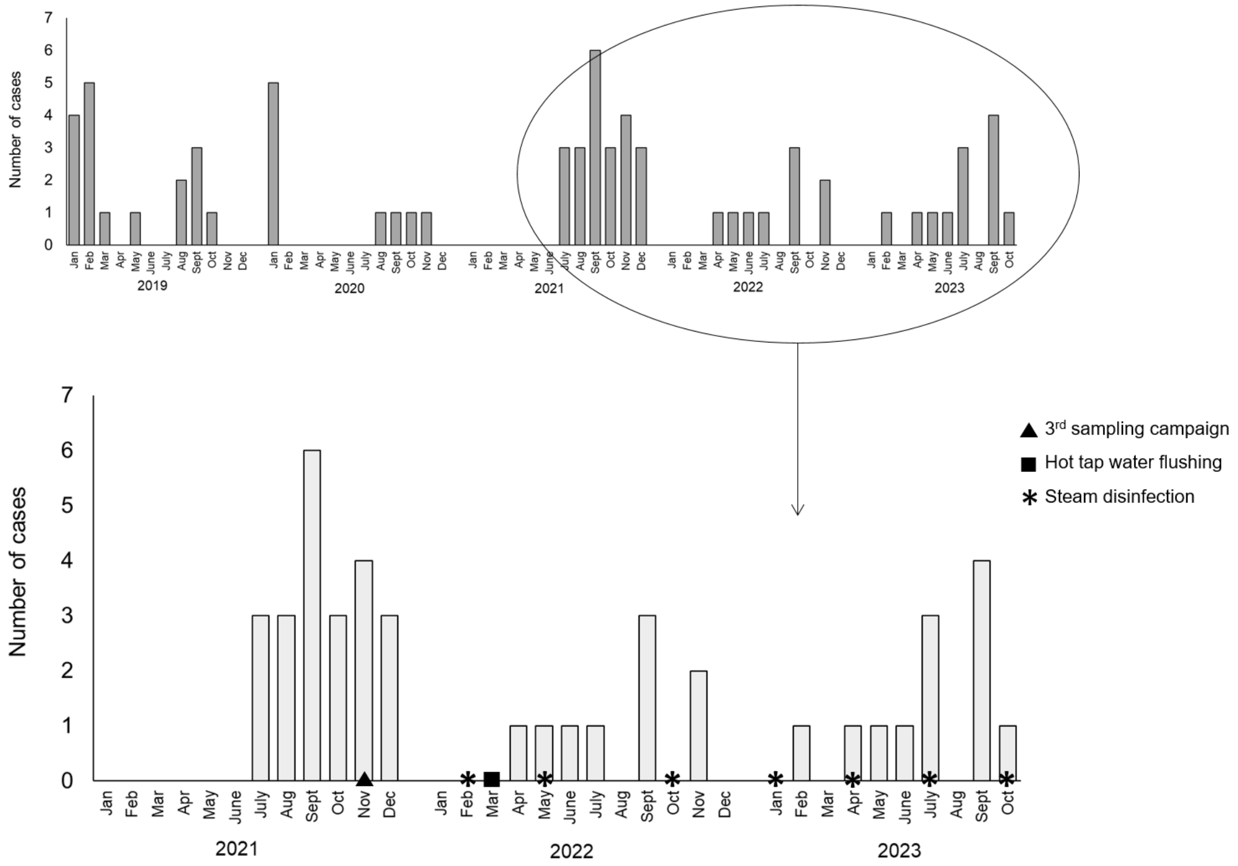

Figure S6. Chronology of nosocomial *S. marcescens* colonizations and infections from 2019 to 2023 in the NICU.

Drain interventions are represented with black symbols. Triangle: third sampling campaign in Nov 2021 (hot tap water flushing (n = 4), steam disinfection (n = 3) and boiling water disinfection (n = 3)). Star: steam disinfection in all the NICU (n = 35) and intermediate care unit (n = 45), except for Feb 2022 (steam disinfection in NICU only) and May 2022 (intermediate care unit only). Square: hot tap water flushing in NICU (n=15).
